# Perceptions of and Barriers to Use of Generic Medications in a Rural African American Population, Alabama, 2011

**DOI:** 10.5888/pcd9.120010

**Published:** 2012-08-30

**Authors:** Keri Sewell, Susan Andreae, Elizabeth Luke, Monika M. Safford

**Affiliations:** Author Affiliations: Susan Andreae, Elizabeth Luke, Monika M. Safford, University of Alabama at Birmingham School of Medicine, Birmingham, Alabama.

## Abstract

**Introduction:**

Using generic medications for chronic diseases provides efficacy similar to that of brand-name medication use, but at a lower price, potentially enhancing adherence. However, previous studies show that disadvantaged people, who may particularly benefit from cost savings, have low trust of generics and increased reluctance to switch to generics. The rural South includes areas of high poverty and minority communities whose members are at high risk for poor health outcomes; however, whether such beliefs exist in these communities has not been reported. We sought to obtain qualitative insight into beliefs about generic medication use among African Americans in the rural South.

**Methods:**

Investigators conducted 4 focus groups with 30 community members from Alabama’s Black Belt area. Transcribed discussions were analyzed and common themes identified.

**Results:**

Participants were primarily unemployed middle-aged women, one-fourth of whom were uninsured and more than half of whom had a high school education or less. Barriers to generic medication use included perceptions that generics are less potent than brand-name medications, require higher doses, and, therefore, result in more side effects; generics are not “real” medicine; generics are for minor but not serious illnesses; the medical system cannot be trusted; and poor people are forced to “settle” for generics.

**Conclusion:**

Although education about generics could rectify misinformation, overcoming views such as mistrust of the medical system and the sense of having to settle for generics because of poverty may be more challenging. Policy makers and providers should consider these perspectives when working to increase generic drug use in these populations.

## Introduction

The bioequivalence of generic and brand medications coupled with substantially lower cost make increasing generic medication use, especially for people with chronic diseases, a health care priority (1-4). Lowering cost can improve adherence; nevertheless, generic medications are underused (1,4).

Multiple factors contribute to generic drug use, including systems-level factors, such as insurance restrictions or state generic substitution laws; provider-level factors, such as physician beliefs or practices (1,5); and patient-level factors, such as consumer perceptions. Negative perceptions of generic medications are higher among the elderly, minorities, and people with low socioeconomic status and health literacy (6,7). Compared with white New Yorkers, African American New Yorkers were more reluctant to switch to generics and had more worries about side effects, medication dependency, and potential harms of generic substitutes (6). Australian patients less frequently used generics for chronic or serious illnesses such as diabetes than for minor illnesses such as allergies (8). Communication with physicians about generics and comfort with generic substitution significantly increased generic use, demonstrating that such barriers could be overcome (9,10).

Beliefs about generic medication safety and efficacy may be particularly relevant in the rural South, a geographic region characterized by high chronic disease prevalence and the highest stroke and coronary heart disease mortality in the United States (11,12). The excess mortality risk is highest for African Americans, who also live in the rural South in higher numbers than in any other rural area of the United States (11). In the Alabama Black Belt, one-third of residents live below the federal poverty line, 1 in 3 African Americans older than 50 years have diabetes, and more than half are overweight or obese (13). Increasing generic medication use in residents of these areas could benefit their health. However, to our knowledge, no studies have reported on perceptions regarding generic medications among rural Southern African Americans with chronic diseases.

In a diabetes intervention trial conducted in Alabama’s Black Belt, we found that many participants used brand-name diabetes medications despite reporting limited income (14,15). We conducted focus groups with area residents who were taking at least 1 medication for chronic disease to better understand beliefs about generic medications.

## Methods

The study was conducted in 2 counties in the rural Alabama Black Belt during 2011. Focus group participants were recruited by using personal contact by community-based staff at a diabetes research program (14,15). These community coordinators serve as local liaisons and have widely established contact networks. Investigators followed up contacts by community coordinators with eligibility screening via telephone who ensured that focus group members were aged 18 or older, Black Belt residents, African American, and taking at least 1 daily medication for hypertension, diabetes, or another chronic disease. The University of Alabama at Birmingham institutional review board approved the study protocol.

Four focus groups totaling 30 participants met in Wilcox and Perry Counties in local community centers. Investigators collected anonymous demographic data at the beginning of each focus group, using simplified forms and verbal instructions. Assisted by a note taker, the moderator used a script (Appendix) to guide the discussion and audio-recorded each group session, which lasted 30 to 60 minutes. Participants received a $10 gift card and dinner as compensation for participation.

Transcripts were imported into NVivo version 9 (QSR International, Cambridge, Massachusetts) qualitative software for data analysis. Two authors (K.S., E.L.) independently reviewed transcripts, identifying major themes or codes. Initial themes were discussed until authors agreed on major themes. Once the codebook was established, the 2 authors coded all transcripts and resolved differing opinions through discussion. By the fourth focus group, no new themes emerged, suggesting saturation had been reached.

## Results

Participants were predominantly middle-aged women (Table 1); 13 (43%) graduated from college, and 4 had less than a high school education. Twenty-two participants (73%) were unemployed, and one-quarter were uninsured. However, only 6 participants had no source of usual care or used the emergency department as their usual source of care. More than 40% of participants took 6 or more medications daily for chronic diseases.

**Table 1:** Characteristics of Focus Group Participants (N = 30), Perry and Wilcox Counties, Alabama, 2011

Characteristic	Perry County Group (n = 7)	Wilcox County Group 1 (n = 9)	Wilcox County Group 2 (n = 6)	Wilcox County Group 3 (n = 8)	Total (N = 30)
Age ≥60 y	5	2	3	2	12
Female	7	9	5	7	28
Graduated from college	3	5	2	3	13
Not employed	7	6	3	6	22
Last visited a doctor, clinic, or hospital <1 month ago	3	4	3	3	13
Has medical insurance	7	8	2	5	22
Regular source of health care^a^	6	9	5	4	24
Takes ≥6 prescription medications daily for chronic diseases	5	5	2	1	13

^a^ Defined as having a personal or private doctor or clinic.

### Theme 1: perceived differences in efficacy

Perceived differences in generic and brand name medication efficacy were commonly expressed (Table 2). In all but 1 group, generics were strongly perceived as being less effective or potent than brand names. Asked to define generics, 1 participant answered, “Not as good as the real medicine” (group B, woman). Other participants stated, “Generic medicine is less potent . . . other medicine is stronger” (group B, woman) and “Name brand is more powerful than the generic” (group D, woman). One participant commented,

**Table 2:** Major Themes Generated by Focus Groups, Perry and Wilcox Counties, Alabama, 2011

Themes	Perry County	Wilcox County Group 1	Wilcox County Group 2	Wilcox County Group 3
Perceived differences in efficacy and side effects	X	X	X	X
Perception that generics are not real medicines		X	X	X
Willingness to take generics for minor but not serious illnesses	X	X	X	X
Trust and medication adherence	X	X	X	X
Perception that, although generics cost less, people of limited means have to settle for generics	X	X	X	X

A lot of times, the doctor gives you a sample and it is the brand name. But when you go to get the medicine, it is too expensive, so you end up with the generic. You can really tell the difference . . . just how effective they are. (group B, woman)

Asked if they would always buy brand medications if they could afford it, several participants in each group responded they would because the brand name was more effective.

Several participants likened this efficacy difference to that between generic and brand-name groceries. One participant noted,

People always say you buy Domino sugar. It’s the best. Don’t buy the cheap brand. Buy Domino and you won’t have to use as much. . . . Domino’s is sweeter. Generic medicine is not as effective as, you know, the real medicine prescriptions, the strength. (group B, woman)

Other participants did not express this belief and shared positive personal experiences with generics: “I take all generics and mine work fine” (group A, woman) and “They just don’t have the name brand, but they do the same” (group D, woman). Several participants received reassurance about generic equivalence from pharmacists or physicians. “We have a good pharmacist . . . and he’ll let you know whether this medicine work[s] or if the generic is cheaper, he’ll say . . . but it do the same thing as the . . . name brand” (group A, woman).

### Theme 2: perceived differences in side effects

Fewer participants expressed concerns about increased side effects as a reason for their hesitancy to use generic medications. Some participants felt that because of their lower potency, generics must be made stronger to be equally effective as the brand-name drugs, leading to more side effects. One participant stated,

Being generic, they have more in it to make it stronger and they might use more synthetic[s] and . . . you might get more of a headache or you might get a little more dizzy or you feel more light-headed. Or sometimes generic can be little bit more of a risk for side effects. It depending on . . . which generic you’re taking. ’Cause everyone cannot take generic. (group B, woman)

Participants related that their friends and family members were also reluctant to use generics. One participant reported,

[My friend] was saying that [the] generic medicine she was taking was not as good as the regular medicine, and she would go back to the doctor to tell him because she was having a bad effect and wanted off the medicine she was taking and she wanted the regular doses of the regular. (group B, woman)

Another participant’s sister believed that the generic would interact negatively with her other medications: “She didn’t want the . . . generic medication[s] . . . because she felt like they would . . . contradict all of the medicines that she was taking easier if they were generic as opposed to being formulary or the brand name” (group B, woman).

Many participants were concerned about possible side effects of both brand and generic medications: “If you read the side effects, you wouldn’t take nothing. . . . All of them have side effects” (group B, woman). Another participant stated, “Some of the side effects, you read that’s on the medicine is more scary than taking it. . . . It scares you a little bit. . . . [I do] not want to take them” (group D, woman). One participant reported that she and her family did not administer her mother’s heart medication because of the listed side effects (group B, woman).

The belief that generics were not suitable for everyone was a theme voiced in all 4 groups: “Some brand medicines are good for some people. . . . Just depends on the person and their system” (group B, woman), and “Yes, it depends on the person. If I can’t take the brand name . . . then sometimes I’ll take the generic. If I can’t take generic, I’ll take the brand name” (group B, woman). Another participant stated, “See, everyone cannot take generics. They have to have a prescribed medication” (group B, woman).

### Theme 3: perceptions that generics are not real medicines

When asked to define brand name medications, participants commonly referred to them as the “real thing” in contrast to their idea of generics (eg, “Something like an off brand from the real thing” [group C, woman], and, “Not the real stuff” [group C, woman]). Another participant said, “If money wasn’t an issue for me, I’d get the real deal” (group D, woman). One participant’s comment was widely supported: “I hope they lower the price of real drugs so people can afford to buy them” (group D, woman).

### Theme 4: willingness to use generics for minor but not serious illness

Participants expressed willingness to use generics for minor illnesses such as allergies and colds but were reluctant to do so for serious diseases. For example, 1 participant would not take generic antihypertensive medications because “High blood pressure is very dangerous. You don’t want to risk your health or your life because of the cost” (group B, woman). When asked about hypothetical generic cancer medications, another participant stated, “My dad has cancer now and I don’t know [about] the cheaper products. I want the best that you know you can get. ’Cause cancer is no joke, that’s serious business” (group B, woman). Another participant echoed this view: “I’ll pay top dollar for that because I know I want to live” (group D, woman).

When asked whether they would choose generic or brand name if they could afford anything, only 1 participant said she would choose generics, “because I am a penny-pincher” (group D, woman). Others said they would choose brand-name medications: “I would go for the best” (group C, woman) and “If money wasn’t a problem, I would go for the brand” (group C, woman).

### Theme 5: trust in the medical system

Many participants reported stopping medications against their doctor’s advice, expressing mistrust of the doctor’s abilities or a hidden financial motive. Many participants noted that “You know more about your body than your doctor does.” One participant stopped her medication because she believed her doctor had inaccurately diagnosed her.

I had one I took myself off of. Thyroid pill. And all other doctors I went to never told me I had thyroid problems. I think that’s something he tried to put on me. And I just stopped taking them because the rest of them didn’t tell me. (group C, woman)

One participant discontinued a medication when she learned that it was an antidepressant, which her doctor had not discussed with her. She viewed this as a reason to mistrust her physician’s prescription choices.

They had me on antidepression medicine. . . . That’s the last thing on my list I be needing. . . . From then on, they gave me a wake-up call. . . . I started looking at any kind of medicine they give me. I look in my book and look up and see what it’s really for. (group C, woman)

Many participants expressed concerns about their relationships with doctors, and some distrusted their doctor’s prescription choices. This distrust stemmed from their belief that doctors were influenced by financial incentives provided by pharmaceutical companies: “Some doctors are pill pushers” (group B, woman) and “The more pills they push, the more money they make” (group B, woman). Another participant hypothesized, “I don’t know if it’s true or not that every prescription that the doctor write, they get a percentage. And so, whether it’s true or not, they sure love to write” (group B, woman), to which another responded, “I think it’s true, too, because they be glad to see the pharmaceuticals come. They invited them in between the patients” (group B, woman).

Group B participants in particular discussed the effect on physician prescribing of the pharmaceutical companies’ use of meals and attractive representatives. A participant noted, “Ever notice those pharmaceutical reps, most of them are nice-looking young ladies or good-looking young men. They dress very well, heels on” (group B, woman). Another responded, “They (pharmaceutical representatives) will wine and dine them to more or less guarantee that the doctor will push their medicine” (group B, woman). Asked whether money influences doctors’ prescriptions, many participants emphatically said yes. “We know doctors go to school. . . . You know they’re well educated. . . . But we know money plays a big part in everything” (group B, woman).

### Theme 6: perception of “having to settle” for generic medications if you are poor

Finances were a substantial part of group discussions. Almost every participant knew that generics were cheaper. However, many participants felt that poor people must “settle” for generic medications. Some associated “settling” with being African American. “The reason being that black Americans take generic ’cause we can’t afford all the name-brand medication,” stated a group B female participant. “A lot of times, we as black Americans go back [to generic medications] because we can’t afford [brand-name medications],” said another group B woman. “They never have nothing cheap. But we settle for that because we’re trying to do the best we can with the fixed income we have.” Some said that generics were mainly geared toward poor people. “Only people that’s worried about that is people that have financial problems. . . . They need the generic brand” (group D, woman). Another group D woman said, “Now, if money wasn’t an issue for me . . . it would never cross my mind about generic.”

Settling for cheaper, second-choice medications was tied to feelings of low-income patients being at the “mercy” of the medical industry. “I think that the doctors and manufacturers use us as guinea pigs to see which ones we will buy the most,” stated one group B woman. “That’s why you are letting the poor people have it. Because we can’t afford it,” said another group B woman. “It’s all about money and the poor people are the ones that have to suffer, and they [drug companies] smile all the way to the bank,” said a third participant (group C, woman). Asked for which illnesses participants would not feel comfortable taking generics, a participant stated, “Cancer medication, I would have to spend the money. I want to live. . . . That’s where they smart, they make that money because they know you want to live” (group D, woman).

Several participants perceived a discrepancy between physician prescribing and insurance coverage, believing that insurance companies forced them to take generics against the doctor’s judgment to save costs: “I think my doctor would have me on all brand-name drugs. But it is the insurance, he put me on the brand name and they say you’ve got to change to generic. So, I take generic ’cause I can’t afford all brand names” (group A, woman). Another participant expressed frustration with insurance companies’ ability to overrule doctors’ choices: “Doctors, they prescribe the name brand drugs. Insurance companies saying generic. So if the doctor is going to be a doctor, why is the insurance company telling the doctor what to do?” (group A, woman).

## Discussion

In this qualitative study, barriers to generic medication use included beliefs about generic medications having lower safety and efficacy and deeper feelings of mistrust of the medical system. Although health education can provide consumers with accurate information regarding the efficacy and safety of generic medications, addressing medication-related beliefs resulting from negative experiences with the medical system, such as mistrust of providers, insurers, or pharmaceutical companies, may be more difficult.

Our findings indicated that residents with chronic disease in the rural Black Belt held some beliefs regarding generic medications and their use similar to those of people living elsewhere, but literature in the United States on this topic is limited. Elderly hospitalized New Yorkers were more likely to hold negative beliefs if they were nonwhite and had low health literacy (6). A national survey found no differences in beliefs about generic medications by race, and approximately 25% of respondents believed that brand-name drugs were more effective than generics (16). Recent reports on this topic have originated primarily from countries other than the United States and revealed themes similar to some of those we described (17); Australians were more willing to switch to generic medications for minor illnesses but not for serious illnesses (8), and Portuguese respondents believed that generics were not as good as brands for serious illnesses (18). The issues of mistrust we described were not reported in other studies of attitudes toward generic medication.

Our study has limitations. Our study population was limited to 2 counties in Alabama’s Black Belt, and participants’ views may not fully represent the views of residents of Alabama and other Black Belt states. Furthermore, although participant race was representative of Wilcox and Perry counties, participants reported higher educational levels and 93% were women, characteristics that may affect generalizability. Although we conducted only 4 focus groups with 30 participants, the lack of new themes emerging by the fourth group suggested that we had reached saturation. By using the community infrastructure of an established research program, we may have enhanced the likelihood of disclosure, because participants were relatives and friends of the community-based research staff. However, these participants also may have been more favorably inclined toward research and the health care system than others in their community. 

We found that African Americans with chronic diseases living in an impoverished rural Southern region of the United States held beliefs regarding generic medications that may contribute to underuse of these medications. Physicians caring for such patients may find these results helpful in guiding their discussion with patients on generic medications. The results of our study also may be useful for policy makers and public health professionals seeking to enhance generic medication use in similar populations.

## Appendix. Moderator Script

**Opening (10 minutes)**

Hello. My name is [moderator name]. Today we would like to have a conversation with you about brand and generic prescription medicines. What we are trying to accomplish before we leave here today is to get a better understanding of how you feel about medications. Are there any questions?

**Respond to participant questions**

Let’s go over some rules. First, let’s all turn off our cell phones so we are not interrupted. So we can keep track of what people are saying, remember that we can only have one person talking at a time. Please do not interrupt someone when they are talking. Also, everything you tell us today will be kept completely confidential. We will summarize the things you tell us and combine it with other focus groups we are giving. One of my jobs today as the moderator is to make sure we discuss all of the issues we planned to discuss. If I ask you questions while you are talking, I’m not being rude; I’m just making sure everyone has a chance to talk and that we discuss all of the issues.

Your opinions are extremely important to us, and we want you to feel free to tell us exactly what you think. Your participation is completely voluntary, and you don’t have to take part in the discussion group. You can withdraw from the discussion at any time, for any reason.

I will be leading the discussion, and [name of assistant] will be taking notes. We will record the discussion. All recorded information is confidential, anonymous, and will be used only for research purposes. The recording will be destroyed at the end of the study.

**Pass out information sheet**

Please take time to read the information sheet. *Go through the sheet as a group.*

**Begin discussion group**

*Ask all participants to introduce themselves. To keep it anonymous, begin recording after introductions.*

Let’s begin.

**Questions 1-5 (45-70 minutes)**

1. What does the term “generic medication” mean to you?

2. How do you feel about taking generic medications?

(probe) How well do you think generic medications work?

(probe) Do you think either generic or brand medications have more side effects, or the same number?

3. Would you switch to a generic medication from a brand medication if your doctor prescribed it?

4. For what kinds of illness (serious, chronic, or minor) would you feel most comfortable taking a generic medication?

5. What are some reasons you would be hesitant to switch from brand to generic medications?

**Closure (10 minutes)**

Did we miss talking about anything else important about generic drugs? Are there any final questions? Thank you for participating in our discussion group today. We are excited to learn about what you think.

## References

[1] Shrank WH , Liberman JN , Fischer MA , Avorn J , Kilabuk E , Chang A . The consequences of requesting “dispense as written”. Am J Med 2011;124(4):309-17. 10.1016/j.amjmed.2010.11.020 21435421

[2] Van Wijk BL , Klungel OH , Heerdink ER , de Boer A . Generic substitution of antihypertensive drugs: does it affect adherence? Ann Pharmacother 2006;40(1):15-20. 10.1345/aph.1G163 16303985

[3] Kesselheim AS , Misono AS , Lee JL , Stedman MR , Brookhart MA , Choudhry NK . Clinical equivalence of generic and brand-name drugs used in cardiovascular disease: a systematic review and meta-analysis. JAMA 2008;300(21):2514-26. 10.1001/jama.2008.758 19050195 PMC2713758

[4] Shrank WH , Hoang T , Ettner SL , Glassman PA , Nair K , DeLapp D . The implications of choice: prescribing generic or preferred pharmaceuticals improves medication adherence for chronic conditions. Arch Intern Med 2006;166(3):332-7. 10.1001/archinte.166.3.332 16476874

[5] Federman AD , Halm EA , Siu AL . Use of generic cardiovascular medications by elderly Medicare beneficiaries receiving generalist or cardiologist care. Med Care 2007;45(2):109-15. 10.1097/01.mlr.0000250293.24939.2e 17224772

[6] Iosifescu A , Halm EA, McGinn T, Siu AL, Federman AD. Beliefs about generic drugs among elderly adults in hospital-based primary care practices. Patient Educ Couns 2008;73(2):377-83. 10.1016/j.pec.2008.07.012 18706784 PMC2739237

[7] Shrank WH , Stedman M , Ettner SL , DeLapp D , Dirstine J , Brookhart AM , Patient, physician, pharmacy, and pharmacy benefit design factors related to generic medication use. J Gen Intern Med 2007;22(9):1298-304. 10.1007/s11606-007-0284-3 17647066 PMC2219782

[8] Babar ZU , Stewart J , Reddy S , Alzaher W , Vareed P , Yacoub N . An evaluation of consumers’ knowledge, perceptions and attitudes regarding generic medicines in Auckland. Pharm World Sci 2010;32(4):440-8. 10.1007/s11096-010-9402-0 20559730

[9] Shrank WH , Cadarette SM , Cox ER , Fischer MA , Mehta J , Brookhart AM . Is there a relationship between patient beliefs or communication about generic drugs and medication utilization? Med Care 2009;47(3):319-25. 10.1097/MLR.0b013e31818af850 19194329 PMC2704338

[10] Huang ES , Brown SES , Thakur N , Carlisle L , Foley E , Ewigman B , Racial/ethnic differences in concerns about current and future medications among patients with type 2 diabetes. Diabetes Care 2009;32(2):311-6. 10.2337/dc08-1307 19017766 PMC2628700

[11] Howard VJ , Kleindorfer DO , Judd SE , McClure LA , Safford MM , Rhodes JD , Disparities in stroke incidence contributing to disparities in stroke mortality. Ann Neurol 2011;69(4):619-27. 10.1002/ana.22385 21416498 PMC3595534

[12] Shuaib FM , Durant RW , Parmar G , Brown TM , Roth DL , Hovater M , Awareness, treatment and control of hypertension, diabetes and hyperlipidemia cholesterol and area-level mortality regions in the Reasons for Geographic and Racials Differences in Stroke (REGARDS) study. J Health Care Poor Underserved 2012;23(2):903-21. 22643632 10.1353/hpu.2012.0045 PMC3771503

[13] ADPH Annual Report 2009. Montgomery (AL): Alabama Department of Public Health; 2009.

[14] Cherrington A , Martin MY , Hayes M , Halanych JH , Wright MA , Appel SJ , Intervention mapping as a guide for the development of a diabetes peer support intervention in rural Alabama. Prev Chronic Dis 2012;9:110053. http://www.cdc.gov/pcd/issues/2012/11_0053.htm. 22239751 10.5888/pcd9.110053 PMC3310146

[15] Andreae SJ , Halanych JH , Cherrington A , Safford MM . Recruitment of a rural, southern, predominantly African-American population into a diabetes self-management trial. Contemp Clin Trials 2012;33(3):499-506. 10.1016/j.cct.2012.02.005 22349456

[16] Shrank WH , Cox ER , Fischer MA , Mehta J , Choudhry NK . Patients’ perceptions of generic medications. Health Aff (Millwood) 2009;28(2):546-56. 10.1377/hlthaff.28.2.546 19276015 PMC2748784

[17] Hassali MA , Shafie AA , Jamshed S , Ibrahim MI , Awaisu A . Consumers’ views on generic medicines: a review of the literature. Int J Pharm Pract 2009;17(2):79-88. 20214255

[18] Figueiras MJ , Cortes MA , Marcelino D , Weinman J . Lay views about medicines: the influence of the illness label for the use of generic versus brand. Psychol Health 2010;25(9):1121-8. 10.1080/08870440903137170 20309776

